# Effects of Aging and Dual-Task Demands on the Comprehension of Less Expected Sentence Continuations: Evidence From Pupillometry

**DOI:** 10.3389/fpsyg.2019.00709

**Published:** 2019-03-29

**Authors:** Katja I. Häuser, Vera Demberg, Jutta Kray

**Affiliations:** ^1^ Department of Psychology, Saarland University, Saarbrücken, Germany; ^2^ Collaborative Research Center on Information Density and Linguistic Encoding, Saarland University, Saarbrücken, Germany; ^3^ Department of Language Science and Technology, Saarland University, Saarbrücken, Germany

**Keywords:** language, aging, context use, sentence comprehension, pupillometry, dual task

## Abstract

Prior studies on language processing in aging have shown that older adults experience integration difficulties for contextually unpredictable target words (as indicated by low cloze probabilities in prior ratings), and that such comprehension difficulties are more likely to occur under more demanding processing conditions (e.g., dual-task situations). However, these effects have primarily been demonstrated for conditions when cloze probability of the linguistic stimuli was very low. The question we asked here was do dual-task demands also impair comprehension when target words provide a good, but not perfect, match with prior context? We used a dual-task design, consisting of a sentence comprehension and secondary motor tracking task. Critical target words were those which were not perfectly predictable based on context (words with a cloze probability of 0.7), as opposed to words that were near perfectly predictable based on context (cloze probabilities of 0.99). As a measure to index online processing difficulty for less expected target words, we took into account participants’ pupil size. Separate mixed effects models were fit for language comprehension, motor tracking, and pupil size, showing the following: (1) dual-task demands led to age-related comprehension difficulties when target words were less expected (as opposed to very highly expected), (2) integration difficulty in older adults was related to cognitive overload as less expected sentence continuations progressed over time, resulting in behavioral trade-offs between language comprehension and motor tracking, and (3) lower levels of working memory were predictive of whether or not older adults experienced cognitive overload when processing less expected words. In sum, more demanding processing conditions lead to comprehension impairments when words are highly unpredictable based on context, as many prior studies showed. Comprehension impairments among older individuals also occur for conditions when words provide a good, but not perfect, match with prior context. Higher working memory capacity can alleviate such impairments in older adults, thereby suggesting that only high-WM older adults have sufficient cognitive resources to pre-activate words that complete a sentence context plausibly, but not perfectly.

## Introduction

Previous studies have demonstrated that aging impairs comprehension of words or sentence fragments that are unexpected based on semantic constraints of prior context (Federmeier et al., 2002, 2010; Federmeier and Kutas, 2005; Wlotko et al., 2012). In addition, such age-related comprehension impairments are more likely to occur under more demanding processing conditions, such as in a dual-task situation when the execution of a secondary task additionally taxes cognitive resources (Tun et al., 2002, 2009; Häuser et al., 2018). The (un)predictability of a word is often assessed by means of cloze probability ratings, where native speakers are asked to continue sentence fragments with the word that comes to mind first. The cloze probability of a word then refers to the proportion of subjects that continued a sentence fragment with that particular word (Kutas and Hillyard, 1984).

However, age-related comprehension impairments for unexpected words have primarily been demonstrated for targets that were very low in cloze probability, in other words, for sentence continuations that were highly unpredictable based on context. Here, we investigated whether aging also affects the comprehension of words that are overly predictable, but do not provide a perfect match with prior context. Specifically, we investigated comprehension of high-cloze items (mean: 0.70; range: 0.11–0.89) and very high-cloze items (mean: 0.99; range: 0.92–1.00). Our main question was whether older adults also experience comprehension difficulties for those target words that do not perfectly match with semantic context.

Many book chapters and review studies describe comprehension of speech and language as the one cognitive faculty that withstands major age-related changes, or even improves with age (see Hedden and Gabrieli, 2004; Glisky, 2007; Burke and Shafto, 2008). Other studies, however, have lately questioned this view, by demonstrating that aging impairs comprehension of words that are unexpected based on prior context. These studies have shown that older adults often only show facilitation (indicated by reduced N400s, an ERP component that normally indexes integration difficulty; Kutas and Hillyard, 1984) for target words which are most highly expected based on a sentence context, whereas younger adults also show facilitation for words that are unexpected, but share semantic feature overlap with the expected words (Federmeier et al., 2002).

For example, when presented with a biasing sentence context such as *They wanted the hotel to look more like a tropical resort, so along the driveway they planted rows of…*, younger adults not only showed facilitation for the target word *palms* (the noun with the highest cloze probability for that sentence) but also for the noun *pines*, which is contextually unexpected (cloze <0.05) but shares semantic features with the target. In older adults, however, there was only facilitation for the most highly expected completion *palms*, whereas the older adults’ ERP response to the semantically related *pines* was the same as for another unexpected control word (*tulips*).

Subsequent studies have added to these findings, by demonstrating, for example, that older adults’ ERP responses do not reliably differentiate between high- and low-typicality exemplars of a semantic category (Federmeier et al., 2010). Thus, when understanding words in discourse, older adults appear to be insensitive toward gradual differences in semantic fit, so that words that are unexpected, but semantically related to highly expected targets, do not experience a processing advantage as seen in younger adults.

In addition to these findings from ERPs, a large body of behavioral research has shown that age-related difficulties with processing unexpected linguistic input are exacerbated by more demanding conditions. Among such conditions are, for example, situations when the speech signal is acoustically degraded (Pichora-Fuller et al., 1995; Tun, 1998; Tun et al., 2002; Benichov et al., 2012; for review, see Tun et al., 2012), or when a secondary task has to be performed at the same time (Wingfield et al., 1985; Tun et al., 1991, 2002; Häuser et al., 2018). According to these studies, older adults rely strongly on contextual constraints during more demanding processing conditions, and experience disproportionate integration difficulties when target words do not match with prior context, or when no semantic context is available that could facilitate processing of upcoming words (Wingfield et al., 1991; Pichora-Fuller, 2008; Lash et al., 2013). For example, in studies that masked auditory speech by noise, older adults’ performance in word recognition often declined dramatically when target words were presented without a supporting discourse context, or when they were embedded in context fragments, which provided very little coherent information (or none at all; Wingfield et al., 1991; Tun and Wingfield, 1994; Tun et al., 2002, 2009). As soon as target words were presented within a coherent semantic context, however, older adults’ performance improved and became similar to that of younger adults.

In dual-task studies, in turn, older adults were sometimes disproportionally affected by unexpected or unpredictable semantic input when situational demands required the execution of a secondary task (Tun et al., 1991, 2002, 2009; Häuser et al., 2018). For example, older adults remembered spoken text passages more poorly in a dual-task situation when the passages had no coherent semantic context and instead were presented with different levels of semantic degradation or scrambling (Tun et al., 2002). When contextually coherent text passages had to be recalled, however, older adults’ performance was similar to that of younger adults.

A common finding in many of these dual-task studies is that older adults adapt to dual-task demands by means of task trade-offs, in other words, by maintaining performance in one of the tasks by neglecting the other task (Kemper et al., 2010, 2014). A study by Becic et al. (2010), for example, demonstrated that older adults’ performance in a primary story-retelling task dropped substantially as soon as a secondary task was introduced that required driving of a computer-simulated car. Other studies have linked task trade-offs in older adults’ dual-task performance specifically to increased difficulty of the linguistic stimuli, for example, in conditions of higher propositional density or grammatical complexity of sentences (Tun et al., 1991; Kemper et al., 2009), or when words cannot be plausibly integrated with prior context. A prior dual-task study from our lab (Häuser et al., 2018), for example, showed that when presented with implausible sentences that mismatched with prior expectations, older adults traded off language comprehension against secondary task motor tracking, by focusing on language comprehension and neglecting tracking.

Thus, the picture that emerges from ERP and behavioral studies is that older adults have difficulty comprehending words that are unexpected based on context, and that such comprehension difficulties are exacerbated by more demanding processing conditions (e.g., dual-task demands). The question that obviously arises is where do these age-related impairments stem from?

Past research has identified three key variables that may account for cognitive impairments in old age: declining processing speed (Salthouse, 1994, 1996), working memory (Salthouse, 1990; West, 1996), and context updating (Braver et al., 2001; Braver and Barch, 2002). Interestingly, according to studies on age differences in language comprehension, these three cognitive variables may also be crucial in predicting age-related impairments in comprehending unexpected linguistic input.

The speed of processing theory explains cognitive aging primarily as slowing in the speed with which mental processes can be executed (Salthouse, 1996). According to this account, age-related difficulties with processing less expected words are primarily due to cognitive slowing, for example, because of age-related delays in spreading activation of lexical-semantic features (Howard et al., 1986; Balota and Duchek, 1988; Balota et al., 1992). Indeed, a previous study by Wlotko and Federmeier (2015) showed that contextual facilitation for unexpected but semantically related words (e.g., *pines*) was reduced in younger adults when subjects had less time to encode words online (operationalized as a shorter stimulus onset asynchrony in word-by-word presentation). Thus, impairments in processing speed may be one factor that could account for age-related comprehension difficulties when semantic input is less expected.

A second important variable that has been used to describe cognitive impairments in old age is working memory (WM). In younger adults, for example, lower WM capacity has been linked to poorer reading comprehension. More specifically, individuals who read less efficiently may have less residual WM capacity to store the products of reading for later recall (Daneman and Carpenter, 1980, 1983; Turner and Engle, 1989; but see Van Dyke et al., 2014, for a conflicting view). In a similar fashion, WM might be linked to the comprehension of unexpected semantic input. Highly predictable sentence contexts presumably tax WM storage only minimally, because they involve the specific pre-activation of only one or very few lexical-semantic concepts (or words). By contrast, less predictable sentence contexts might involve the weak and more wide-spread pre-activation of multiple lexical-semantic features and/or words (see Federmeier and Kutas, 1999). As such, less predictable sentences might be more taxing for WM, given the information required to keep in storage is considerably greater. And again, there is evidence in the extant literature that this might be the case. In Federmeier et al. (2002), for example, a subset of older adults – those with higher verbal fluency (the ability to produce speeded, thematically appropriate output on demand without repetition) – demonstrated the young-like ERP pattern by showing gradual facilitation for unexpected but semantically related words (*pines*). Yet other work has suggested working memory capacity to be the core component that drives individual differences in verbal fluency (Daneman, 1991). Thus, working memory seems another cognitive component that relates to comprehension of unexpected linguistic input.

Finally, a third potential variable that may influence processing of unexpected language could be impairments in successful activation and updating of context information (e.g., Braver and Barch, 2002). Encountering contextually unpredictable words likely requires an updating mechanism that signals a semantic conflict between prior context and new incoming information, and engages in a late-stage recovery where contextual information is revised and the unexpected word successfully integrated (e.g., Kintsch and Van Dijk, 1978). Unsurprisingly, prior studies have indeed linked age impairments in processing unexpected language to specific impairments in context updating. One study (Haarmann et al., 2005), for example, showed that older adults’ error rates in meaningfulness judgments to unpredictable sentence continuations correlated highly with their performance in the AX-CPT task, a common task that measures context maintenance and updating.

In sum, it seems that individual differences in processing speed, working memory, and the ability to quickly update contextual information appear to mediate the comprehension of unexpected linguistic input. However, it is currently unknown whether one of the three cognitive variables may in fact be more important than others in predicting comprehension impairments in aging. Settling this question was therefore another objective of the present study.

Here, we used a dual-task paradigm, consisting of a language comprehension and continuous motor tracking task, to investigate whether dual-task demands induce comprehension impairments for target words that are not perfectly predictable based on context (cloze probabilities of 0.70: high cloze; vs. cloze probabilities of 0.99: very high cloze). If older adults have difficulty processing not only completely unexpected words but also words that do not provide an ideal match with prior context, we expected that there should be comprehension disruptions for the less predictable high-cloze (but not very high-cloze) sentence continuations. Based on prior studies, we expected that such disruptions should be particularly likely to occur under dual-task demands when resources to integrate less expected input are less readily available (Pichora-Fuller et al., 1995; Benichov et al., 2012).

As an online measure to index cognitive load during processing of expected and less expected words, we used pupillometry (Beatty, 1982; Beatty and Lucero-Wagoner, 2000), a technique that – to our knowledge – has not previously been used to study age-related differences in comprehension of unexpected language. Pupillometry takes advantage of the physiological response of the pupil to dilate under conditions of cognitive load, and has been used to index, for example, processing difficulties during digit recall and arithmetic tasks (Kahneman and Beatty, 1966). More importantly for the present study, however, pupillometry has also been applied to study age-related differences in cognitive load during language processing, for example, word recognition under noise (Zekveld et al., 2011), processing of less expected syntactic structures (Piquado et al., 2010), or comprehension of semantic violations based on context (Häuser et al., 2018).

Our first question was whether older adults would demonstrate comprehension difficulties for predictable, but less expected sentence continuations (high cloze, as opposed to very high cloze) particularly under conditions of divided attention (dual-task situation). Such a finding would extend our knowledge about age-related impairments in comprehending unexpected linguistic input, which has been shown primarily for items that are highly unpredictable (very low cloze probabilities; Federmeier et al., 2002; DeLong et al., 2012; Wlotko and Federmeier, 2015). In hindsight of prior studies demonstrating greater dual-task trade-offs among older adults, a related question concerned the role of behavioral adaptations in older adults. How would older adults adapt to encountering less expected sentence continuations under dual-task demands, and what would their pupillary response say about the allocation of attentional resources and possibly cognitive overload (Häuser et al., 2018)?

Our second question concerned the role of individual differences, in hindsight of studies that illustrated moderating effects of processing speed, working memory, and context updating. Which cognitive factors, over and above any effects of age, are more predictive of age-related difficulties in processing less expected semantic input?

## Materials and Methods

### Participants

The sample consisted of 32 younger adults (mean age = 25 years, age range = 19–34 years) from Saarland University and 30 older adults from the Saarbrücken community (mean age = 72 years, age range = 65–78 years), who were compensated with cash at a rate of EUR 10 per hour. All participants were native speakers of German who had normal or corrected-to-normal hearing and no history of neuropsychological and/or psychiatric disorders. The study was approved by the Computer Science ethics committee at Saarland University. All study procedures were in line with the Helsinki Declaration on human subject testing. Informed written consent was obtained from all participants.

All subjects were administered a battery of three cognitive tests to assess individual differences in processing speed, working memory capacity, and context updating. Tests consisted of the Digit Symbol task (adapted from Wechsler, 1955), the forward Counting Span task (see Conway et al., 2005, for review), and a modified version of the AX continuous performance task (AX-CPT task; adapted from Schmitt et al., 2014).

**Figure 1:**
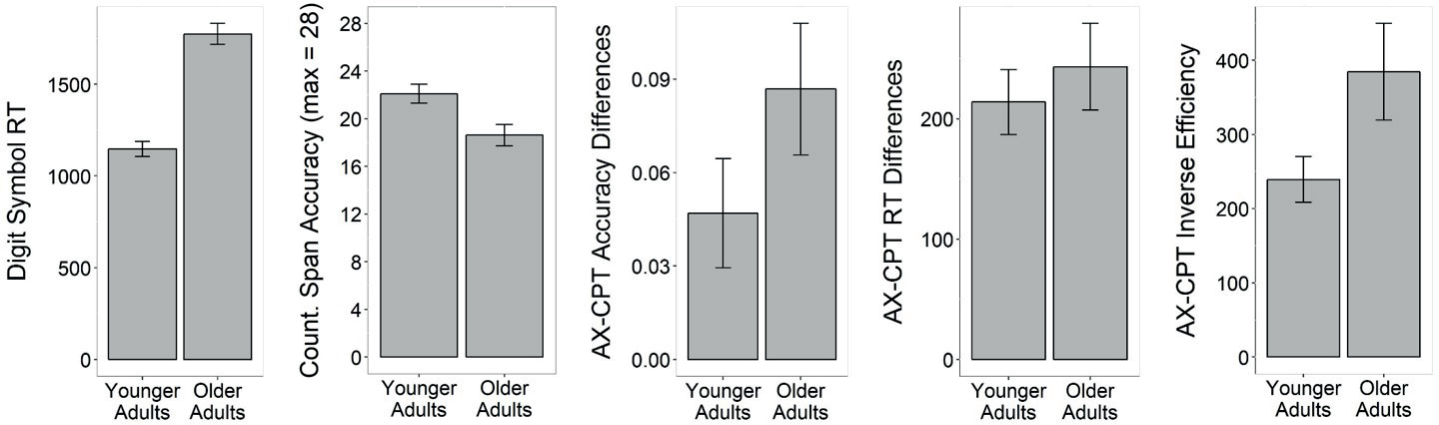
Group-wise performance (±subject-weighted error bars) in the Digit Symbol, Counting Span, and AX-CPT tasks.

Figure 1 displays group-wise reaction times from correct trials in the Digit Symbol task and the total number of correctly remembered trials in the Counting Span task. For the AX-CPT, we computed subject-wise difference scores (for accuracy and RT separately) between context-dependent (harder) and context-independent (easier) trials, as well as inverse efficiency scores to set accuracy and RT in relation (Bruyer and Brysbaert, 2011; Vandierendonck, 2017).

For accuracy differences scores, we subtracted accuracy in context-dependent (harder) trials from those in context-independent (easier) trials. For RT difference scores, we subtracted RTs in context-independent (easy) trials from those in context-dependent (hard) trials. For inverse efficiency scores, we divided subject-wise average reaction times in context-dependent trials by their average accuracy in context-dependent trials, and subtracted from that value subject-wise reaction times in context-independent trials divided by their average accuracy in context-independent trials (i.e., [RT_dependent_/accuracy_dependent_]–[RT_independent_/accuracy_independent_]). Greater inverse efficiency scores indicate a greater cost balance between context-dependent and context-independent trials, and thus, lower levels of context updating.

As can be gleaned from Figure 1, older adults consistently performed more poorly than younger adults across all cognitive tests. Reaction times in the Digit Symbol task were significantly higher for older compared to younger adults, *t*(52.54) = 8.97, *p* < 0.001. There was also a significant main effect of age in the total number of correctly remembered trials for the Counting Span task, *t*(57.14) = −2.89, *p* < 0.01, with older adults remembering fewer trials correctly than younger adults (19 vs. 22, out of 28 in total). There were no significant effects of age in either the AX-CPT reaction time cost score (difference in RT between context-dependent and context-independent trials), *t*(53.30) = 0.65, *p* > 0.05, or the accuracy cost score (difference in accuracy between context-independent and context-dependent trials), *t*(55.95) = 1.45, *p* > 0.05. For AX-CPT inverse efficiency scores, the main effect of age was barely significant, *t*(40.10) = 2.02, *p* = 0.05.

Table 1 displays a Pearson product correlation matrix of the relationship between the Digit Symbol reaction times, total number of correct trials in the Counting Span task, and AX-CPT accuracy, RT difference, and inverse efficiency scores for the whole sample of subjects. As the table shows, none of the cognitive measures significantly correlated with one another, with three exceptions. First, the Counting Span and Digit Symbol tasks showed a significant negative correlation with one another that was moderate in size (*r* = −0.38, *p* < 0.01), suggesting that individuals with higher working memory were faster in speed of processing. Second, accuracy differences scores in the AX-CPT task negatively correlated with the counting span task (*r* = −0.25, *p* < 0.05), indicating that individuals with higher working memory were also better in context updating. Third, inverse efficiency scores in the AC-CPT task also correlated negatively with the counting span task (*r* = −0.31, *p* < 0.05).

**Table 1:** Pearson product correlation matrix of the three cognitive tests.

	1	2	3	4	5
1. Digit symbol (RT)	1.00				
2. Counting span	−0.38^**^	1.00			
3. AX-CPT accuracy difference	0.01	−0.25^*^	1.00		
4. AX-CPT RT difference	0.25	−0.12	0.12	1.00	
5. AX-CPT inverse efficiency	0.21	−0.31^*^	–	–	1.00

Significance codes: *p < 0.05; **p < 0.01.

### Design and Procedure

**Figure 2:**
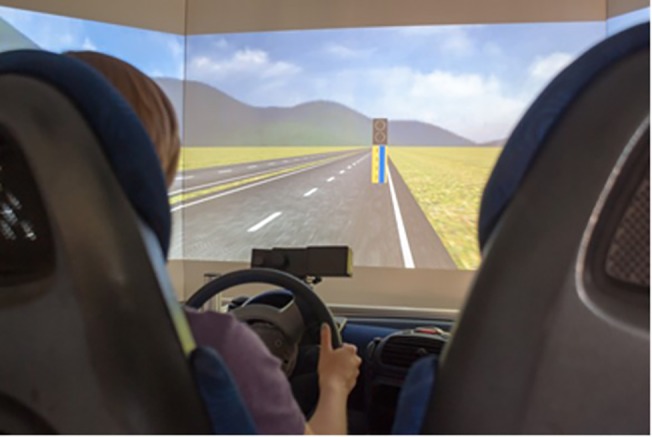
The continuous motor tracking task (“ConTRe” task; Mahr et al., 2012).

Our dual-task design employed a sentence comprehension and a continuous motor tracking task. In the sentence comprehension task, participants performed semantic meaningfulness judgments to sentences varying in cloze probability, by verbally responding to the question “Was the sentence meaningful and correct?”, while the experimenter coded their responses. The continuous motor tracking task was operationalized as a driving task (ConTRe Task; Mahr et al., 2012). Two vertical color bars moved laterally on a road at a continuous speed (see Figure 2). By turning a steering wheel, participants could control one of the bars (the blue one), whereas the other bar (the yellow one) was continuously moving (controlled by the computer). Participants were instructed to continuously track the yellow bar with the blue one, in order to keep the distance between the two bars minimal at all times.

In the single-task condition, participants performed motor tracking or sentence comprehension in isolation. In the dual-task condition, participants performed the motor tracking and sentence comprehension tasks at the same time. Overall, the experiment consisted of six experimental blocks: two dual-task blocks, two language-only blocks, and two motor tracking-only blocks, sequenced as single tracking, single language comprehension, dual comprehension and motor tracking, dual comprehension and motor tracking, single motor tracking, and single language comprehension.

Pupil size was measured continuously throughout the experiment, and participants were instructed to move their heads as little as possible during the duration of one experimental block. The experimental session took place in a completely dark room, shielded from day light. The wrap-around projection screen from the motor tracking task was the only source of illumination that participants directly faced, and its luminance remained constant throughout the experimental session (note that the mountain-road display and the vertical bars shown in Figure 2 remained on the screen even when participants were performing language comprehension with no motor tracking).

### Materials

Stimulus materials for the sentence comprehension task consisted of auditory versions of the 48 low-surprisal items from Häuser et al. (2018). An example set of stimuli is given below, showing (in bold) the target words which varied in cloze probability:

Am portugiesischen Strand baute der kleine Junge einige sehr schöne **Sandburgen** mit seinen Freunden. (*At the beach of Portugal, the little boy built some very nice **sand castles** with his friends*).

Letzte Woche strich unser freundlicher Nachbar für uns den **Zaun** vor seiner Garage. (*Last week, our friendly neighbor painted the **fence** in front of his garage for us*).

An einem Sommerabend lernte der Medizinstudent für seine **Prüfung** im Garten. (*In a summer night, the medical student studied for this **exam** in the garden*).

For all target words, cloze norms were obtained from two groups of younger (*n* = 20) and older adults (*n* = 18), who did not participate in the main experiment. Cloze probability was defined as the proportion of individuals to complete the sentence with that particular word. As observed in prior studies (e.g., Federmeier et al., 2002), cloze ratings in younger and older adults were qualitatively and quantitatively similar (*M*_Younger Adults_ = 0.86, *M*_Older Adults_ = 0.79), even though older adults tended to be more variable in their cloze ratings than younger adults (*SD*_Younger Adults_ = 0.13, *SD*_Older Adults_ = 0.25). However, these differences were lexical rather than semantic in nature (e.g., for a sentence which could be completed in “chimney” or “smokestack” (in German, “Kamin” vs. “Schornstein”), younger adults tended to use “smokestack,” whereas older adults tended to use “chimney”). Based on the group-wise ratings, items in each age group were median-split into two groups, a group of sentences with high-cloze probability (cloze ratings ≤0.89 in both groups, mean 0.7; range 0.11–0.89), and a group of sentences with very high-cloze probability (cloze ratings ≥0.90; mean 0.99; range 0.92–1.0). Means and standard deviations for high and very high-cloze ratings in the two age groups are presented in Table 2. Prior to the experiment, all experimental items were synthesized using the MARY text-to-speech system (Schröder and Trouvain, 2003) and manipulated so that the duration of the target word was identical across sentences.

**Table 2:** Cloze ratings for high and very high-cloze sentences, depending on age group.

	Younger adults	Older adults
High cloze	0.77 (0.10)	0.66 (0.23)
Very high cloze	0.98 (0.03)	1.0 (0)

Standard deviations in parentheses.

### Apparatus

Participants were seated behind the steering wheel, with the driving projection screen surrounding them and the eye-tracker installed at the top of the dashboard. The driving environment was projected on three large panels positioned in a 180° angle around the steering wheel using OpenDS 3.0 (opends.de) software. Both tasks (motor tracking and language comprehension) were controlled from a separate computer using Experiment Builder.^1^ Auditory items for the language comprehension task were presented through Creative Gigaworks T20 speakers. Eye-tracking data were collected using the remote mode (no chin rest) of the Eye-link 1000 Plus system (SR-Research, Ontario, Canada), sampled at a rate of 250 Hz. Participants were instructed to fixate on the lateral bars on the motor tracking display and to move their head as little as possible. Before each block, eye movements were calibrated using a nine-point grid. Eye-tracking data were collected for both eyes. The cognitive test battery (Counting Span task, Digit Symbol task, and AX-CPT task) was controlled by E-Prime and presented on a Dell Ultra Sharp U2515H computer screen, using a screen resolution of 2,560 × 1,440 pixels.

## Results

Our dependent variables included response accuracy in the language comprehension task (a binary variable), steering deviations in the motor tracking task, and pupil size. Fixed effects were cloze probability (high vs. very high-cloze probability), task condition (single vs. dual), and age group (younger vs. older adults). We constructed separate linear mixed effects models for each dependent variable as implemented in the lme4 library (Bates et al., 2015; version 1.1-18) in R (R Development Core Team, 2016; version 3.4.3). Models were fit with random intercepts for subjects and items, and random slope parameters for all corresponding within-subject and within-item effects warranted by the design (i.e., a fully maximal random effects structure; see Barr et al., 2013). Note that the present study was a follow-up on the low-surprisal stimuli used in Häuser et al. (2018), where high- and low-surprisal was fully experimentally crossed over items, but cloze probability among the low-surprisal items was not fully crossed. Hence, by-item random slopes for cloze probability were not considered here. Categorical predictors were centered in order to reduce multicollinearity. In the case of non-converging models, each model was simplified progressively using the *least variance* approach until convergence was achieved (see Barr et al., 2013; for guidelines). Higher order interactions were followed up with planned model splits between younger and older adults. *p* were estimated using the Satterthwaite degrees of freedom method, as implemented in the R package *lmerTest*.

### Response Accuracy in Sentence Comprehension

The final model for response accuracy included fixed effects for age group, task condition, and cloze probability, as well as by-subject and by-item random intercepts. Convergence issues with this model did not allow for random slope adjustments.

**Figure 3:**
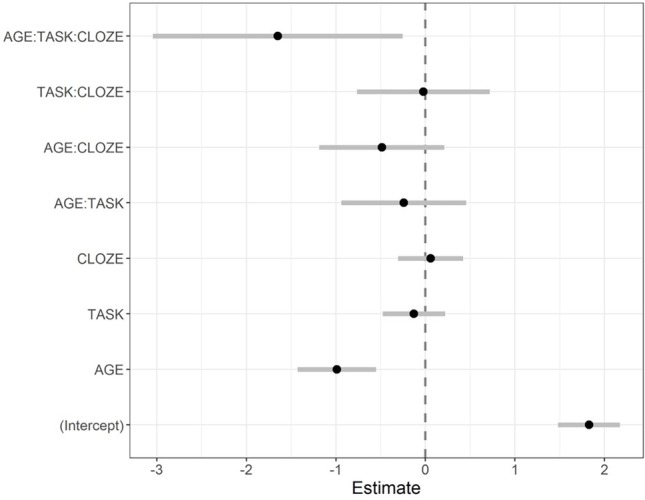
Fixed-effect parameter estimates of the response accuracy model and corresponding 95% confidence intervals.

**Figure 4:**
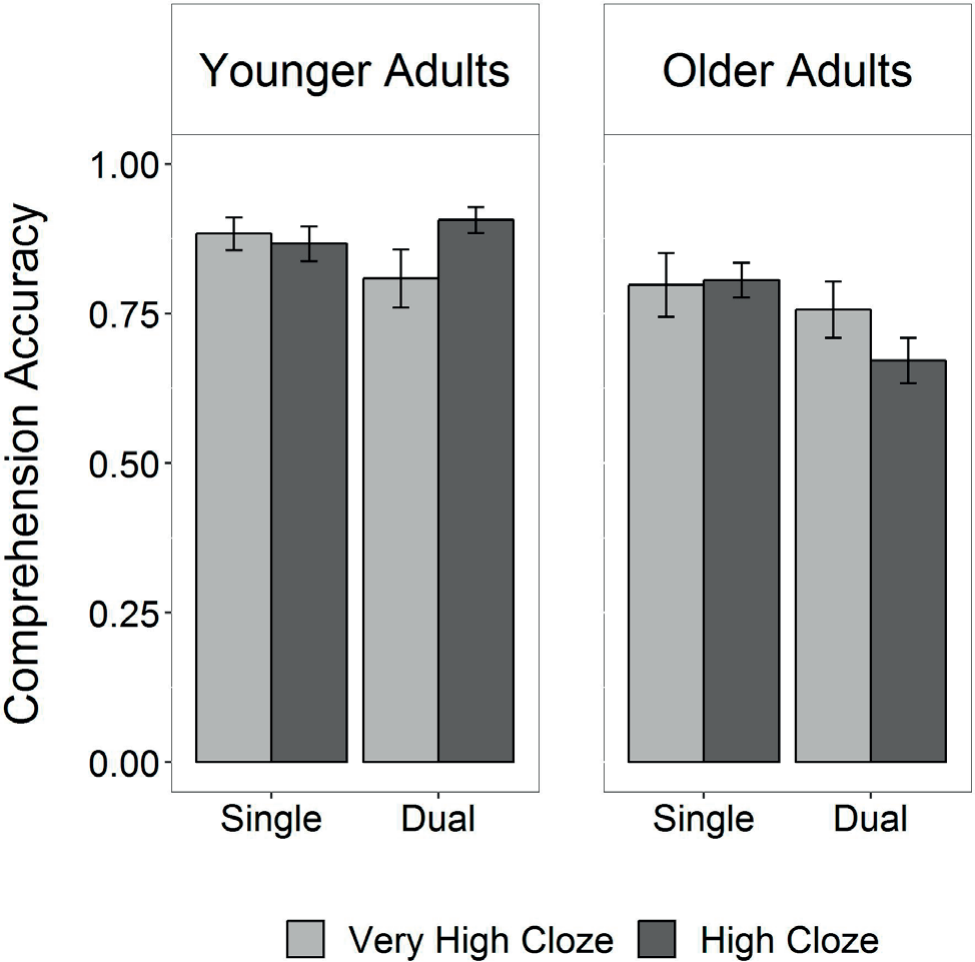
Response accuracy (±subject-weighted error bars) in sentence comprehension, depending on age group, task condition, and cloze probability.

We found a three-way interaction between age group, cloze probability, and task condition (*b* = −1.65, *SE* = 0.71, *z* = −2.32, *p* < 0.05; see Figure 3, for coefficient plot with parameter estimates of the model). Figure 4 shows a bar plot of the accuracy data. Of key interest here is whether cloze probability interacts with age group particularly in the dual task, which would provide statistical support for the claim that older adults show comprehension impairments for less expected sentence continuations when task demands are increased. Thus, to further probe this three-way interaction, individual models were fit testing the interaction between cloze probability and age group separately in each task condition. Indeed, only the model for the dual-task condition showed a significant interaction between age group and cloze probability (*b* = −1.25, *SE* = 0.50, *z* = −2.49, *p* < 0.05; interaction in single-task model: *b* = 0.12, *SE* = 0.60, *z* = 0.20, *p* > 0.5). As another set of model follow-ups showed, the group effect was only significant in high-cloze sentences (*b* = −1.53, *SE* = 0.36, *z* = −4.29, *p* < 0.001), but not in very high-cloze sentences (*b* = −0.32, *SE* = 0.46, *z* = −0.70, *p* > 0.1).

Thus, analysis of the accuracy data suggested that dual-task demands reduced older adults’ comprehension accuracy for less expected sentence continuations, whereas younger adults’ comprehension performance was high for both highly expected and less expected sentence continuations.

### Steering Deviations in the Motor Tracking Task

Note that the effects of cloze probability on motor tracking can only be investigated in dual-task trials (only those contain language comprehension *and* motor tracking). Thus, for the purposes of this study, we focus on statistical analysis of dual-task trials only (see Häuser et al., 2018, for analysis of the single- vs. dual motor tracking data). Raw steering deviations were coded as negative and positive values (indicating left- and right-sided deviations), so we squared all values to obtain a final measure. Prior to analysis, steering deviations were trimmed for outliers at 3 SD above and below each individual’s mean in the single- and dual-task conditions. The final model for squared steering deviations contained random intercepts for subjects and items, as well as by-item random slopes for age group.

**Figure 5:**
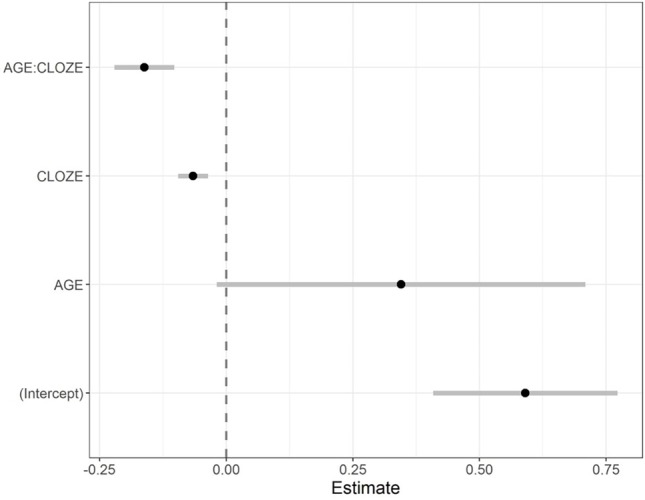
Fixed-effect parameter estimates (±95% confidence intervals) of the model for steering deviations.

**Figure 6:**
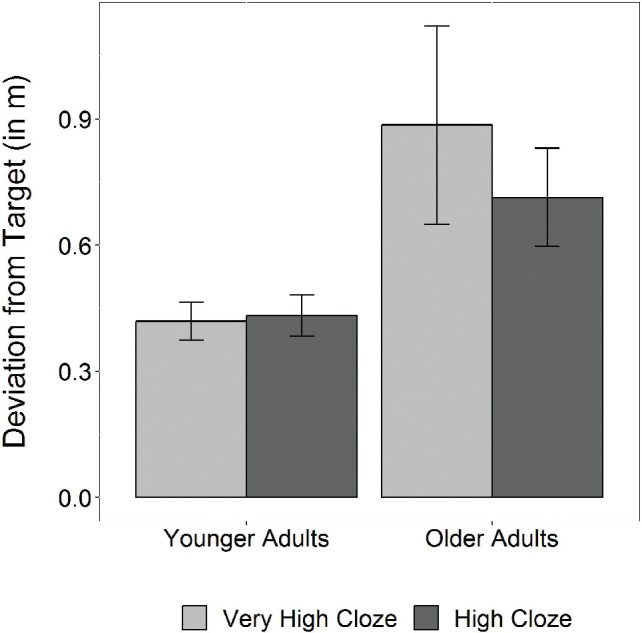
Steering deviations in the continuous motor tracking task (±subject-weighted error bars) during dual-task performance, depending on age group and cloze probability.

The model showed a significant interaction between age group and cloze probability in the dual-task condition (*b* = −0.16, *SE* = 0.03, *t* = −5.35, *p* < 0.001; see Figure 5, for coefficient plot). For bar plot, see Figure 6. Figure 6 indicates relatively low steering deviations for younger adults, with no visible differences between high- and very high-cloze sentences. By contrast, older adults’ steering deviations seemed to be relatively higher for very high-cloze items. To verify these observations, we performed a model split to investigate effects of cloze probability separately in younger and older adults. As expected, there was only a significant main effect of cloze probability in older (*b* = −0.16, *SE* = 0.03, *t* = −4.75, *p* < 0.001) and not in younger adults (*b* = 0.02, *SE* = 0.01, *t* = 1.29, *p* > 0.1), demonstrating relatively lower steering deviations for high-cloze sentences and higher steering deviations for very high-cloze sentences.

Thus, analysis of the motor tracking data showed stable performance in younger adults, irrespective of cloze probability of the linguistic stimuli, but worse performance in older adults, where steering deviations were lower during dual-task comprehension of less expected sentence continuations. By contrast, steering deviations were higher during comprehension of very highly expected sentence continuations (very high-cloze targets).

### Pupil Size During Sentence Processing

**Figure 7:**
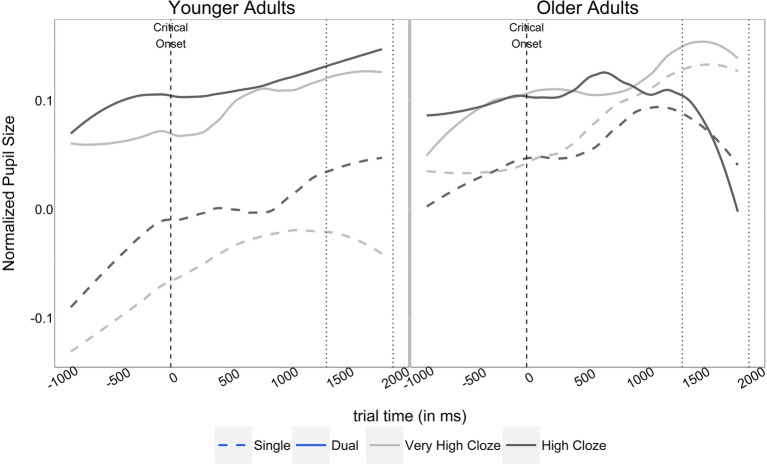
Normalized pupil size (±90% confidence bands) over time, depending on age group, task condition, and cloze probability. Dotted lines indicate the critical region for statistical analysis of the pupil size data.

Eye-tracking data in both age groups were preprocessed by excluding missing data points (blinks, tracker loss) as well as gaze positions that fell outside of the screen. We normalized raw pupil size values (Beatty and Lucero-Wagoner, 2000; Van Gerven et al., 2004; Piquado et al., 2010) by subtracting the average pupil size per participant from the raw pupil values for that participant, and dividing the resulting value by 2.5 times the subject-wise standard deviation. Finally, we excluded trials for which normalization had resulted in a loss of more than 25%. Altogether, this resulted in a loss of 12.06 and 17.27% from the younger and older adults’ data, respectively. Visual inspection of the normalized pupil size data over time (see Figure 7) suggested a gradual decrease in pupil size for older adults during later stages of high-cloze sentence comprehension, particularly during the dual-task condition in the time interval between 1,400 and 2,000 ms after critical word onset. Statistical analyses were then performed for observations in this critical time window, which is compatible with prior studies using pupillometry in general (e.g., Piquado et al., 2010; Experiment 1), and pupillometry in language processing more specifically, including work from our group (e.g., Piquado et al., 2010; Häuser et al., 2018, Experiment 2). To assess gradual changes in pupil diameter, we added time (in ms) as a scaled variable to the model (time-locked to the onset of the critical word). Thus, there were four predictors in the model for pupil size: age group, task condition, cloze probability, and time. The four-way interaction in the model, as well as the three-way interactions between task condition, cloze probability, and time, and between task condition, age group, and time, were not significant so they were excluded from the fixed effect structure (*χ* ^2^(3) = 4.77, *p* = 0.19). The final model included random intercepts for subject and items, as well as maximal random slope adjustments for subjects and items. We also included a random effect for eye, nested within subjects.

**Figure 8:**
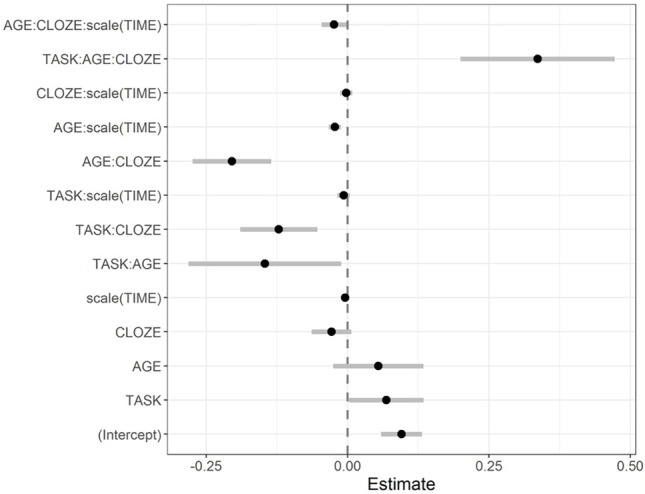
Fixed-effect parameter estimates of the pupil size model and corresponding 95% confidence intervals.

There were significant interactions between age group, cloze probability, and time (*b* = −0.02, *SE* = 0.01, *t* = −2.14, *p* < 0.05; see Figure 8, for coefficient plot) and between age group, task condition, and cloze probability (*b* = 0.33, *SE* = 0.07, *t* = 4.84, *p* < 0.001). To investigate the source of these complex three-way interactions, we ran follow-up models in which we split subjects by age group.

The model for younger adults showed a significant interaction between task condition and cloze probability (*b* = −0.28, *SE* = 0.06, *t* = −5.0, *p* < 0.001). Figure 7 (left panel) demonstrates that effects of cloze probability were large for younger adults during single-task performance, with larger pupil sizes for high-cloze sentences than for perfectly predictable very high-cloze sentences. By contrast, pupil sizes during the dual-task condition suggested relatively smaller effects of cloze probability. These observations were verified by another model split, which demonstrated that the cloze effect was larger in magnitude for single-task trials (*b* = 0.20, *SE* = 0.05, *t* = 4.2, *p* > 0.001) than it was for dual-task trials (*b* = 0.08, *SE* = 0.03, *t* = 2.57, *p* > 0.01).

The model for older adults, in turn, showed a significant three-way interaction between task condition, cloze probability, and time (*b* = −0.04, *SE* = 0.02, *t* = −2.05, *p* < 0.05). Whereas pupil sizes in older adults seemed to globally decrease during the critical time window, Figure 7 (right panel) indicates that this decrease was relatively steeper and more pronounced for the less expected targets during dual-task performance (as opposed to the highly predictable targets). Thus, to further probe this interaction, we performed another model split and computed separate follow-up models for single vs. dual-task performance. In line with the pattern observed from the graph, only the model for the dual-task condition showed significant effects of cloze probability over time (*b* = −0.03, *SE* = 0.01, *t* = −2.79, *p* < 0.01; single-task model: *b* = 0.00, *SE* = 0.01, *t* = 0.23, *p* > 0.1), with pupil sizes showing a relatively steeper decrease over time for less expected high-cloze sentence continuations (*b* = −0.03, *SE* = 0.01, *t* = −3.06, *p* < 0.001) than those of highly predictable, very high-cloze continuations (*b* = −0.01, *SE* = 0.01, *t* = 1.29, *p* > 0.1).

In sum, statistical analysis of the pupil size data in the critical time window suggested two primary findings of interest: first, younger adults showed large effects of cloze probability during single-task performance, but diminished effects of cloze probability during the dual task. Second, older adults demonstrated a gradual decrease in pupil size during processing of less expected (as opposed to highly predictable) items, which, when considered with their reduced performance on the comprehension questions, can be interpreted as a signal of reduced attentional effort and overload (see Zekveld et al., 2011; for similar findings in pupillometry).

### Individual Differences

To assess the source of inter-individual variability, which may explain the age-related decrease in pupil size associated with comprehending the less predictable high-cloze sentences in the dual task, we examined the relationship between the cognitive measures obtained for each participant and the pupillometric measure of interest. Our key interest was to identify which one(s) of the three previously identified variables (processing speed, working memory, and context updating) are crucial in explaining cognitive load and comprehension impairments among older adults. To this end, we computed pupil difference scores by subtracting the subject-wise pupil average for high-cloze sentences from the subject-wise average for very high-cloze sentences during the dual task and the critical region time interval that was used for statistical analysis of the pupil data. Corresponding to the experimental finding that pupil size decreased in older adults for the less expected high-cloze (as opposed to very high-cloze) items, average differences scores were larger for older (*M* = 0.06) than for younger adults (*M* = −0.04). Subject-wise difference scores were then entered as dependent variable into a stepwise hierarchical regression. We used the *step*() function in R to compare an age-only model (with the continuous variable age as only predictor) to a full model that accounted for the effects of age, working memory capacity (WM capacity; the maximal number of correct items in the Counting Span task), processing speed (average RTs in the Digit Symbol task), and context updating (accuracy and RT differences in the AX-CPT task), excluding interactions between these factors or their interactions with age. Model fit was assessed forward and backward, i.e., new predictors were only added to the model if they improved model fit, and irrelevant predictors were discarded after each step if they became irrelevant by the adding of new variables.

The best fitting model (determined by means of the Akaike information criterion) contained age and WM capacity as only predictors. In this model, only WM capacity (and not age) was a significant predictor of pupil size difference (*b* = −0.01, *SE* = 0.004, *t* = −2.75, *p* < 0.01; effect of age: *b* = 0.00, *SE* = 0.00, *t* = 1.36, *p* > 0.1). Overall, this model accounted for a large portion of variance in pupil size (Multiple *r* ^2^ = 0.20, Adjusted *r* ^2^ = 0.18). Model diagnostics to assess fit returned no influential cases; all residuals had a Cook’s distance <1. The relative importance of each predictor in the model was assessed by means of the package *relaimpo*, which estimates the relative importance of predictors by accounting for whether they were entered first or last into the model. The results indicated that the relative contribution of age to the final proportion of variance accounted for in the model (0.20 in total) was much lower than the one for WM capacity, 0.06 and 0.14, respectively. Standardized betas of the two predictors confirmed this pattern (*b*_age_ = 0.17, *b*_WM capacity_ = 0.35).

**Figure 9:**
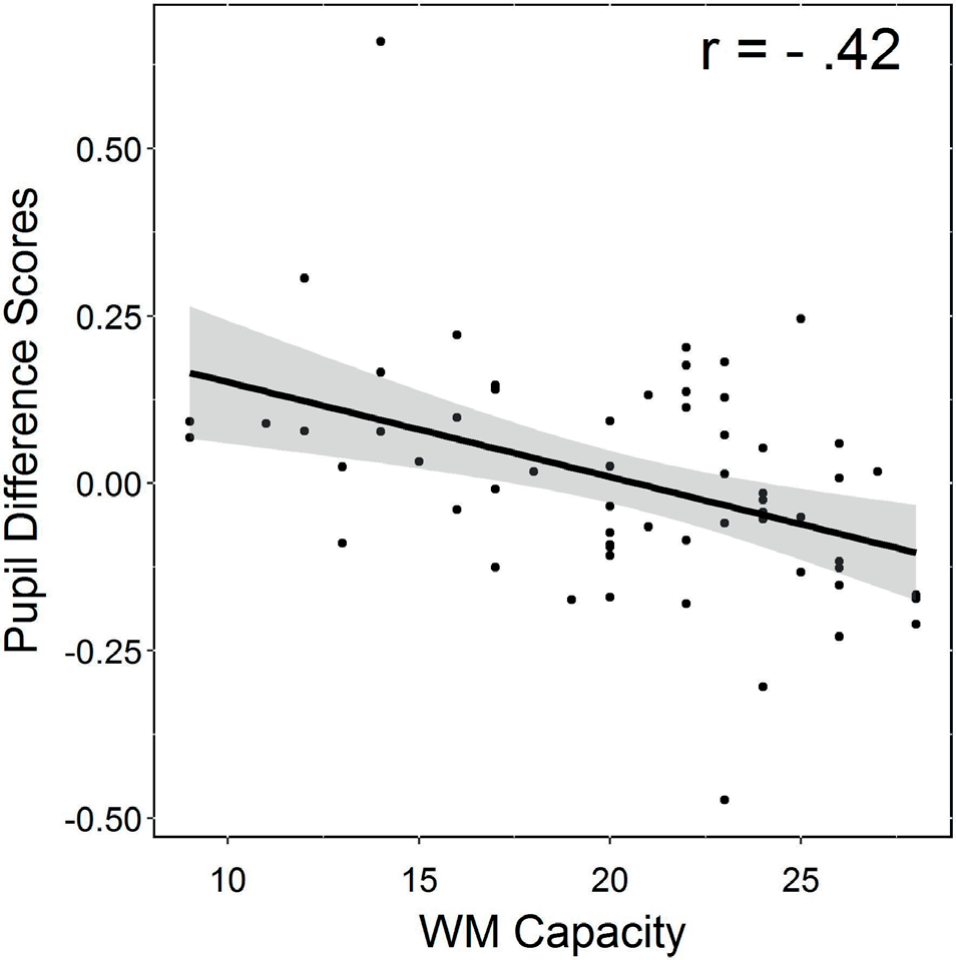
Correlation plot (±90% confidence bands) showing the relationship between WM capacity and pupil size.

Thus, even when age was accounted for, WM capacity and pupil size showed a significant negative correlation, that is, for individuals with higher WM capacity, the decrease in pupil size (signaling overload) associated with dual-task processing of less expected sentence continuations was smaller than the one for individuals with lower WM capacity (see Figure 9, for a correlation plot of the effects of WM capacity on pupil size). Thus, the pupil size decrease for less predictable high-cloze (as opposed to very high-cloze) sentences observed in older adults seemed primarily related to declining WM capacity.

## Discussion

We used a dual-task paradigm, consisting of a primary language comprehension and secondary motor tracking task, to investigate how demanding processing conditions affect older adults’ comprehension of target words that are either highly predictable based on context or slightly less predictable. Prior studies had demonstrated age-related comprehension impairments primarily in conditions when target words or sentence continuations were completely unexpected based on prior context (very low cloze probabilities; Federmeier et al., 2002, 2010; Federmeier and Kutas, 2005).

Here, we investigated whether older adults also experience comprehension difficulties when target words are predictable, but do not provide a perfect match with prior context (targets with high-cloze probability, cloze ~ 0.7; as opposed to targets with very high-cloze probability, cloze ~ 0.99). Our expectation was that comprehending the slightly less predictable high-cloze target words would be more difficult for older adults, especially during dual-task demands when attentional resources to quickly integrate less expected input are not readily available.

We measured participants’ task performance in the language comprehension task (accuracy of meaningfulness judgments for high- vs. very-high-cloze sentence continuations), and their performance in the secondary motor tracking task (which required continuous tracking of a moving target by turning a steering wheel). As a measure of online sentence processing, and to assess fine-grained changes in cognitive load as target sentences unfolded over time, we measured participants’ pupil size (e.g., Beatty, 1982; Beatty and Lucero-Wagoner, 2000; for use in older adults, see Piquado et al., 2010; Zekveld et al., 2011).

Our first question was whether dual-task demands would exacerbate age-related processing difficulties for sentence continuations that do not perfectly match with prior context, and, if so, what behavioral adaptions we would find in the older subject group. As hypothesized, and in line with prior literature (e.g., Tun et al., 2002, 2009; Pichora-Fuller, 2008), age-related difficulties during processing of less expected target words were more likely to emerge under dual-task demands, when cognitive resources were more taxed due to the secondary motor tracking task. Consistent with a prior study from our group (Häuser et al., 2018), where task trade-off between language processing and motor tracking emerged when target words were completely unexpected and implausible based on context, our results here show a similar trade-off when target words are plausible, but not perfectly predictable based on context.

Specifically, during the dual task, older adults showed lower comprehension accuracy for high-cloze (as opposed to very high-cloze) targets, but at the same time maintained relative stable motor tracking performance when processing these items. In addition, their pupil size revealed that during online listening of the slightly less expected target words, older adults demonstrated a gradual decline in pupil size, a finding that has previously been interpreted as increasing loss of attentional focus and cognitive overload (Zekveld et al., 2011). In contrast, older adults’ comprehension accuracy was good for highly predictable sentence continuations (i.e., very high-cloze items), even though during comprehension of these sentences, there were steady declines in continuous motor tracking.

Taken together, these results suggest a complex set of behavioral adaptations and trade-offs in older adults that appeared to be driven by task demands on the one hand, and cloze probability of the stimuli on the other hand: For highly expected sentence continuations (cloze probability ~ 0.99), older adults maintained high comprehension accuracy with declining motor tracking; for slightly less expected sentence continuations (cloze probability ~ 0.7), older adults showed increasing loss of attentional focus during online sentence processing with declining comprehension accuracy, but stable motor tracking.

These data corroborate a large body of research showing that older adults demonstrate greater dual-task costs than younger adults (Verhaeghen and Cerella, 2002; Verhaeghen et al., 2003), and that older adults adapt to dual-task demands by relevelling their task priorities and trading off one task against another (e.g., Li et al., 2001; Kemper et al., 2009; Becic et al., 2010; Häuser et al., 2018). Our results also support earlier studies using ERP, by suggesting that older adults have difficulty integrating words that are contextually unexpected (Federmeier et al., 2002).

Crucially, however, our findings expand our knowledge about language comprehension impairments in old age. Whereas prior studies had mostly demonstrated such impairments for conditions when words were completely unpredictable, our findings here suggest that aging and dual-task demands impair the comprehension of words that deviate from contextual expectancies only minimally and in nuances (as soon as target words were not highly predictable). These comprehension impairments are then manifested in cognitive overload during online processing and subsequent comprehension difficulties. Language comprehension impairments in healthy aging may thus be more far-reaching than most current accounts assume (e.g., Burke and Shafto, 2008; Shafto and Tyler, 2014).

So what brings on these age-related difficulties in comprehending less predictable words under adverse processing conditions? One obvious explanation could be that older adults simply do not have sufficient cognitive capacities to split attention between two tasks and quickly integrate less expected target words with prior contextual information. Aging is generally known to bring along substantial declines in executive functions of cognition, declines which have previously been linked to difficulties with some aspects of language comprehension, for example, the comprehension of syntactically complex sentences (e.g., Kemtes and Kemper, 1997; Stine-Morrow et al., 2000). Importantly, individual differences in executive functions among older adults are at least part of the explanation for the current set of findings (see below, for discussion).

An alternative, though not mutually exclusive, possibility could be fundamental differences in how younger and older adults encode sentential context during online processing of language. For example, many prior studies suggested that older adults more strongly rely on message-level, top-down strategies during language encoding, as opposed to bottom-up, sensory encoding strategies, which are less reliable and often impaired in aging (e.g., Stine-Morrow et al., 1996, 2006). This preferential reliance on top-down processing has been linked to compensatory adjustments that effectively try to “make up” for age-related declines in sensory aspects of speech processing. However, it has also been related to older adults’ life-long experience with language that potentially enriches their mental representations in verbal memory and semantic storage (Hedden and Gabrieli, 2004). The consequence of older adults’ greater reliance on message-level, top-down processing strategies could be that older adults encode only the most gist-wise, high-level information from language – one that is somewhat impoverished of semantic nuances. Consequently, only target words that match with this high-level information show facilitated integration, whereas other less likely alternatives (e.g., targets with a slightly lower cloze probability) do not.

A similar point is evident in studies on hearing research (e.g., Wingfield et al., 2005; Tun et al., 2012), where individuals with poorer hearing acuity often show declining performance in downstream tasks (e.g., in speech recall) that might be related to poorer encoding of speech online. For example, individuals with mild hearing loss were less likely to correctly recall the first two words of a three-word sequence than individuals with normal hearing maybe because the effort it took them to even recognize the words came at the expense of correctly encoding the stimuli and rehearsing them for greater recall (the “Effortfulness Hypothesis”; see Rabbitt, 1968; Wingfield et al., 2005). Similar effects could also be at work during dual-task demands when processing resources of older listeners are more taxed because they have to split attention between two tasks. This splitting of attention could possibly lead to a less semantically elaborate mental representation of contextual information in working memory, where only gist-wise, high-level information is maintained. As a consequence, only targets that match with this high-level, less semantically elaborate representation are facilitated, whereas other, more nuanced fits with context are not.

In contrast to the older group, our findings for the group of younger adults suggest no behavioral adaptations or difficulties for less expected linguistic input, even though younger adults’ pupil indicated graded processing effort, depending on single vs. dual-task demands and contextual predictability of the sentences. Specifically, younger adults’ pupil size was greatest during dual-task comprehension of high-cloze items, smaller for dual-task comprehension of very high-cloze targets, and smallest for single-task processing of high-cloze and very high-cloze items.

This replicates findings from ERPs in younger adults, demonstrating that language users are reliably sensitive to small nuances in cloze probability and show graded patterns of cognitive effort during comprehension of linguistic load (e.g., Federmeier and Kutas, 1999). To our knowledge, this study is the first to demonstrate such fine-grained and nuanced differences in linguistic load by using pupillometry. Therefore, pupillometry is a promising tool to add to investigations of electrophysiology and behavior that have previously been used to index integration difficulties during language processing.

Our second major question for the purposes of this study concerned the role of individual differences. Previous studies had identified three cognitive components that might play a key role in processing unexpected language, namely working memory capacity, processing speed, and contextual updating (Federmeier et al., 2002; Haarmann et al., 2005; Wlotko and Federmeier, 2015). It is an ongoing issue in psycholinguistics whether these cognitive variables are equally important in moderating language comprehension, or whether some are, in fact, more important than others.

Our data here indicate that general working memory capacity among older listeners (as indexed by the Counting Span task), but not processing speed or context updating (as measured by the Digit Symbol and AX-CPT tasks, respectively), was related to the gradual decline in pupil size that we found in older adults when they were listening to less expected sentence continuations. In other words, older adults’ increasing loss of attentional focus for unexpected sentence continuations was especially pronounced for individuals with lower working memory capacity, but it was reduced for older individuals with higher working memory capacity.

The comparatively greater role of WM capacity suggests that successful comprehension of unexpected semantic input is largely driven by cognitive resources. Whereas high-WM individuals might pre-activate not only the most expected but also slightly less expected words or features during sentence processing, low-WM individuals, in contrast, might only have sufficient resources to pre-activate the most highly predictable semantic concepts, but fail to activate others. Hence, only low-WM older adults show comprehension impairments and cognitive load for the less expected high-cloze (as opposed to very high-cloze) sentences. Our data converge with the results from a study by Federmeier et al. (2002), which suggested that only older adults with higher verbal fluency (a construct related to WM capacity) showed facilitated integration for target words that shared semantic features with the most highly expected noun. In sum, age-related comprehension impairments for unexpected words are largely driven by limitations in WM capacity, maybe because low-WM individuals only manage to pre-activate highly predictable words based on context, but not others.

The present data indicate that context updating and processing speed are less crucial (when compared to WM capacity) in determining age-related comprehension difficulties for unexpected language. The lack of an effect for processing speed, in particular, is remarkable, because recent debates have been centered around the importance of this variable. For example, some researchers have hypothesized that, if given enough time, older adults may be able to overcome initial integration difficulties with unexpected language, because over time, activation for even less expected words or semantic features can start to accrue (e.g., Wlotko and Federmeier, 2015). According to such accounts, older adults might merely be slower in pre-activating less predictable semantic features (also see Balota et al., 1992). Our data speak against a crucial role for processing speed, even though the nature of our auditory language comprehension task might have supported such a role, at least more so than the word-by-word presentation rate that many ERP studies used. Ultimately the lack of an effect for processing speed could be due to the fact that our auditory sentences were presented at a relatively moderate speed and that subjects completed the experiment without any time constraints (e.g., had ample time to respond to the comprehension questions). It is possible that speeded response conditions, or the use of time-compressed speech would have yielded different results with respect to that variable (Wingfield et al., 1985; Wingfield, 1996; Tun, 1998). The comparatively greater role of WM capacity, in turn, could be due to the dual-task nature of our experiment, a set-up which is designed to strain cognitive resources more than single-task experiments where attention does not have to be divided between multiple tasks.

## Conclusion

Prior studies had suggested that aging impairs comprehension of unexpected semantic features or words, especially under adverse processing conditions such as dual-task demands. It was unknown, however, whether dual-task demands also affect the comprehension of words that are overly predictable, but do not provide an ideal match with prior context. The study presented here clearly suggests that this is the case: under dual-task demands, older adults showed comprehension impairments and processing disruptions for target words that did not perfectly match with prior semantic context. Regression analyses showed that these age-related impairments were largely driven by declining working memory capacity in older adults. This suggests that only high-WM older adults have sufficient cognitive resources to pre-activate words or concepts that complete a sentence context sensibly (but not perfectly). Our findings add to the growing body of research demonstrating the influence of aging on language comprehension, and the role that non-linguistic cognitive functions play in processing of speech and language.

## Data Availability

The datasets generated for this study are available on request to the corresponding author.

## Author Contributions

The study was conceived and designed by VD. JK and KH contributed to the data collection. The data were analyzed by KH. KH, VD, and JK were involved in the interpretation of the results. KH wrote the manuscript in its current form. VD and JK contributed to revising it. All authors approved the current version and agreed to be accountable of all aspects of the work.

### Conflict of Interest Statement

The authors declare that the research was conducted in the absence of any commercial or financial relationships that could be construed as a potential conflict of interest.

## Footnotes

^1^sr-research.com
